# Impacts on oral health attitude and knowledge after completing a digital training module among Swedish healthcare professionals working with older adults

**DOI:** 10.1186/s12913-024-10639-3

**Published:** 2024-02-07

**Authors:** Maria Snogren, Kristina Ek, Maria Browall, Irene Eriksson, Ulrika Lindmark

**Affiliations:** 1 School of Health Sciences, University of Skövde, Högskolevägen, 541 28 Skövde, Box 408, Sweden; 2 Research School of Health and Welfare, Jönköping University, Jönköping, Sweden; 3 Department of Nursing, School of Health and Welfare, Jönköping Academy for Improvement of Health and Welfare, Jönköping University, Jönköping, Sweden; 4 Affiliated with the Department of Oncology, Institute of Clinical Sciences, Sahlgrenska Academy, University of Gothenburg, Gothenburg, Sweden; 5 Department of Health Sciences, Karlstad University, Karlstad, Sweden

**Keywords:** Health care training, Municipality care, Fundamentals of care, Oral health care

## Abstract

Oral health care is essential, and digital training may influence healthcare professionals’ attitudes to and knowledge of oral health. The aim, therefore, was to evaluate the impact on attitudes to and knowledge of oral health after using a digital training module among Swedish healthcare professionals working within a municipality-run healthcare service for older adults. A secondary aim was to explore the healthcare professionals’ experiences of using the digital module. The study comprised a survey of healthcare professionals (registered nurses (RNs), assistant nurses, and care assistants) caring for older adults in a municipality in Sweden. Pre-post-tests were conducted to evaluate the outcomes for attitudes to and knowledge of oral health and of their experiences of completing the digital training module in oral health. These were statistically explored by comparing differences between the pre-post-tests, while the open-ended questions were analysed with qualitative content analysis. The findings of this study indicate that healthcare professionals had similar perceptions of their attitudes to and knowledge of oral health both before and after the digital training module in oral health. The study also indicates that healthcare professionals experienced that it is easier to perform practical oral health care after completing the digital training. The results also show that healthcare professionals value oral health knowledge and that the digital training module was easy to use and to disseminate knowledge throughout the municipality. The findings have implications for developing, implementing, and promoting healthcare professionals’ attitudes to and knowledge of oral health and in using a digital training module in combination with practical exercises in oral health in municipality health care.

**Supplementary Information:**

The online version contains supplementary material available at 10.1186/s12913-024-10639-3.

## Background

Oral health is multi-faceted and influences a person’s ability to act in daily life, such as speaking and eating, but also to convey a range of emotions through facial expressions [1]. Good oral health can promote general health and well-being, can lead to a more independent life for older adults and a reduced need for care [2], and can be cost-effective for the person and society by preventing any further progression of dental pathology [3]. Oral diseases and conditions such as dental caries, periodontitis, and fungal infections can be prevented among older adults in municipal health care (home health care and nursing homes) provided by healthcare professionals (registered nurses, assistant nurses, and care assistants) [4, 5]. Nevertheless, previous research has shown that older adults’ resistance and professionals’ lack of time, and also aids [6], routines, guidelines [7, 8], violation of integrity [9–11], resources [6] and healthcare professionals’ attitudes and knowledge [12] have all presented barriers to oral health care, which also affects the quality of oral health care provided to older adults in need of care. K Edman and I Wårdh [12] conclude that oral care is not a high priority in a stressful work environment where many tasks are to be carried out, and also suggest the promotion of oral care training and theoretical education about oral health and disease among healthcare professionals. Therefore, skills development in oral health care is needed for healthcare professionals working within older adults’ care to prioritise and effectively perform Fundamentals of Care (FoC) related to oral health care [13]. The Swedish National Board of Health and Welfare [14] also states that oral health care is a part of healthcare professionals’ work duties and needs to be prioritised and provided in skills development programmes. Healthcare professionals must have tools such as the knowledge to achieve FoC as it relates to oral health care in practice. Care delivery needs to move from a series of tasks to a coordinated, integrated, person-centred mode of healthcare delivery [13, 15, 16].

Skills development for healthcare professionals allows them to maintain a high quality of care, and healthcare professionals need knowledge about oral health to achieve good FoC in relation to oral health care [13, 15, 16]. Skills development for healthcare professionals, such as training about oral health, can be challenging to implement; often, only a few undergo the training, and it can be challenging to disseminate such knowledge within an entire working group [17]. Skills development is also not prioritized, as resources must be taken from other tasks to facilitate this [18]. Studies show that healthcare professionals who receive skills development in oral health are able to provide good oral health care [19] and improve the oral health of older adults (persons 65 years or older) [20]. Continuing professional learning is crucial in the development of skills relating to the FoC [21], e.g., those related to oral health. New ways of working, with the support of new technology and increased collaboration, can contribute to skills development for healthcare professionals, where digital forms of education/skills development have been suggested [14].

Digital training modules are able to quickly disseminate standardised knowledge across an entire working group and can be made easily accessible and flexible relating to the time and place in which the learning activities occur, thus increasing opportunities for learning and for refreshing that learning [22]. Digital training modules have previously been evaluated in training in oncology with positive results related to experiences of completing the training [23]. Therefore, a digital oral healthcare training module could be one solution to develop healthcare professionals’ skills and improve their knowledge about oral health. Studies evaluating oral health knowledge and healthcare professionals’ attitudes and knowledge relating to oral health have demonstrated the importance of training programmes to increase both skills development and knowledge of and attitudes to oral health care [12, 24, 25]. Therefore, the aim was to evaluate the impact on attitudes to and knowledge of oral health, using a digital training module among Swedish healthcare professionals working within a municipality providing health care for older adults. A secondary aim was to explore the healthcare professionals’ experiences of using the digital module.

### Specific research questions

- Are there any differences in attitudes to and knowledge of oral health and care before and after a digital training module in oral health?
- Are there any group differences in attitudes to and knowledge of oral health and care regarding workplace, age, and number of years in the profession?
- How do healthcare professionals experience completing a digital training module in oral health?

## Methods

The study employed a mixed-methods design, according to A Tashakkori and JW Creswell [26] and all of the COREQ (Consolidated criteria for Reporting Qualitative research) elements were considered when conducting the study. Data were collected through: (a) a quantitative sample completing a questionnaire about attitudes to and knowledge of oral health questionnaire (AKO) and their experiences of completing the digital training module in oral health, and (b) a qualitative sample of free-text answers to the open-ended questions from the questionnaire to explore their experiences of completing the digital training module in oral health.

### Participants and data collection

**Fig. 1:**
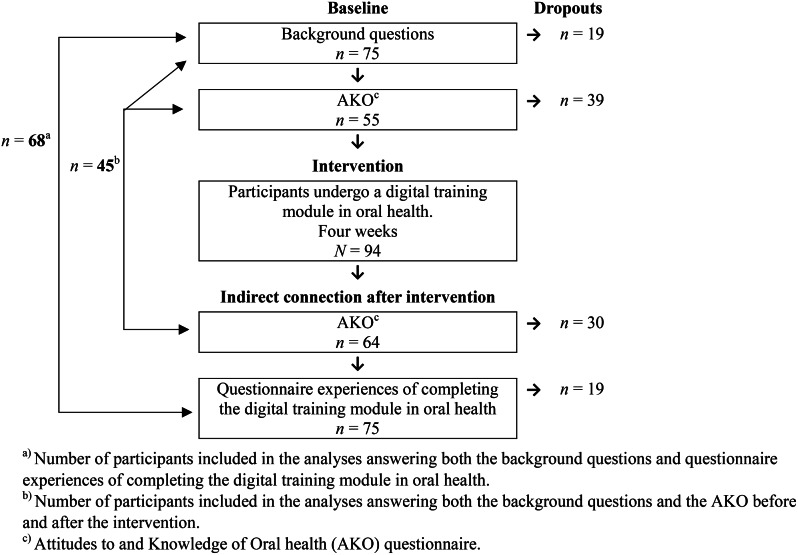
Data collection procedure

The study comprised a survey of all permanently employed healthcare professionals (registered nurses (RNs), assistant nurses, and care assistants) (*N* = 94) working in healthcare settings (nursing homes and short-term care and home care) in an urban municipality in the western part of Sweden (5730 inhabitants) in the spring of 2022. The municipality has the overall responsibility for providing nursing care in both home healthcare services and municipality-run nursing homes. After information about the study was provided to the head of the department for the included municipality, the telephone and e-mail contact details of potential participants were sent to one of the researchers (first author, MS) after permission was granted by the healthcare professionals. An information letter was sent via e-mail to all healthcare professionals who met the inclusion criteria of being permanently employed healthcare professionals who can read and understand Swedish. The rationale for including only those who could read and understand Swedish is that being able to read and speak Swedish is a condition of employment for those working as healthcare professionals in the care of older people in order to cope with their work, and in requesting their participation in the study and understanding information about the project. The data collection procedure is described in Fig. 1. The healthcare professionals were asked to complete the questionnaires before and directly after completing the digital training module.

It was mandatory for the participants (*N* = 94) to undergo and complete the digital training module in oral health as it forms a part of the municipality’s current quality improvement work. Answering the questions was voluntary; therefore, it was not possible to conduct non-response analyses. Of the 94 participants, 45 (48%) answered both background questions and AKO before and after, and 68 (72%) answered both the background questions and the questionnaire relating to their experiences of completing the digital training module in oral health, which formed the results.

### Digital training module in oral health

The digital training module in oral health was developed in collaboration with RNs from the research group, dentists from the Swedish Centre for Older Persons Dental Care (in Swedish, centrum för äldretandvård, CÄT), and an educator from the Swedish company, Lära Nära (in Swedish). Lära Nära provides the web-based training module on an internet-based platform accessed via a browser. It requires no special software, and is easy to navigate and understand. The module adopts both a nursing and dental care perspective. The module contains five sections: (1) Introduction to oral health, (2) Oral health, (3) Caries, gum, and periodontitis, (4) Oral health and general health, and (5) Palliative care. The digital training module in oral health was designed so that the participants could complete it individually, at their own pace, and with the ability to take breaks and repeat sections. The sections can be reviewed in any order, and the completion of the entire module takes about two hours. The participants were provided access to the module via Lära Nära online and they were registered using their workplace-related e-mail addresses. Each participant’s first and last name and e-mail address (linked to the workplace) were sent to one of the researchers (the first author, MS) who was responsible for administering user accounts for the training module.

### Questionnaires

The questionnaires were answered within the digital training modules (both before and after the training) in oral health, on a web-based platform (provided by Lära Nära).

#### Attitudes to and knowledge of oral health questionnaire (AKO)

The AKO assesses healthcare professionals’ attitudes to and knowledge of oral health and advocates 13 items in a short form in three subject groups: (1) Attitudes to oral hygiene, three items graded on a Likert scale from 1 (never) to 5 (always), lower scores signify a better attitude to oral health (min 3 to max 15); (2) Implementation possibilities, four items on a Likert scale from 1 (never) to 5 (always), high scores signify better implementation possibilities (min 4 to max 20); and (3) Knowledge of importance, six items on a Likert scale from 1 (unimportant) to 5 (important), high scores signify knowledge importance (min 6 to max 30). These three subject groups are considered to be three key concepts and have acceptable validity and reliability for assessing healthcare professionals’ attitudes to and knowledge of oral health [27, 28]. The questionnaire is described in Appendix 1.

#### Questionnaire: experiences of completing the digital training module in oral health

The research group developed a questionnaire to gather the participants’ experiences of participating in a digital training module in oral health. The questionnaire contains eighteen statements about their experiences of undergoing the digital training module, graded on a four-degree Likert scale, from 1 (‘a very low extent’) to 4 (‘a very high extent’), and seven additional open-ended questions with free-text answers related to the eighteen questions in the questionnaire. The questions covered areas such as functionality, the purpose of the training, knowledge levels, and advantages and disadvantages of the digital design. No validation was performed in relation to the questionnaire.

### Data analysis

The quantitative data were entered and analysed in International Business Machines Corporation (IBM) Statistical Package for the Social Sciences (SPSS) [29] version 27. Descriptive statistics were used to illustrate demographic characteristics. The Wilcoxon signed-rank test was used to compare the AKO responses before and after the digital training module, in total and within each sub-group. The Mann-Whitney *U* test was used to compare differences between each sub-group before and after the digital module. For the descriptive analysis of the questionnaire gathering experiences of completing the digital training module in oral health, the scores for ‘a very low extent’ and ‘a low extent’ were merged due to the small group size and skewed distribution between the scores. Sub-groups included in both analyses were workplace (home care and nursing home, with nursing home also including short-term care for analytical reasons), age (20–44 and 45–67 years), and years in the profession (1.5–18 and 19–47 years) with the cut-off point set at the mean level to create equivalent group sizes. The subgroups of gender, duration of work experience in the profession, and skills development, including work duties and experience of managing oral health care, were excluded from the analysis due to the small group size and skewed distribution between the groups. The significance level for both analyses was set at *p* < 0.05. Descriptive statistics were used to illustrate the participants’ experiences of completing the digital training module in oral health.

To describe healthcare professionals’ perceptions of completing a digital training module in oral health from the qualitative responses in the questionnaire (experiences of completing the digital training module in oral care), conventional content analysis, influenced by H-F Hsieh and SE Shannon [30], was used. The data analysis took place by repeatedly reading the seven open-ended questions with free-text answers to gain an overall view of, and then to reflect upon, the contents. Then the text was read word-by-word to find similarities and differences in the text, and these were sorted into categories. All the authors were involved in all the steps, and the texts were also discussed and reflected upon by all authors to secure trustworthiness [30].

## Results

Participants who were included in the analysis of the AKO (*n* = 45) were mostly women (80%) and assistant nurses (75%) who worked during the daytime (66%) as shown in Table 1. Their ages ranged from 20 to 67 years, with a median age of 38 and a mean age of 44.5 years. Work experience ranged from 1.5 to 47 years, with a median of 13 years and a mean of 18.5 years. More than half (56%) of the participants worked in home care, and less than half (44%) in a nursing home. Almost all participants (91%) considered that oral health care is included in their work duties. About one in five had previously undergone some form of skills development in oral health, and three out of four reported that the workplace management of oral health was ‘good’ or ‘very good’. These figures were similar for those answering questions about their experiences of completing the digital training module (*n* = 68).

**Table 1:** Partcipants charactaristics

		AKO ^a)^ before and after *n* (%)	Experiences of completing the digital training module in oral health *n* (%)
**Total**		45 (48)	68 (72)
**Gender**	Men	9 (20)	12 (18)
	Women	36 (80)	56 (82)
**Age**	20–44	24 (53)	33 (49)
	45–67	21 (47)	35 (51)
**Profession**	Nurse	3 (7)	5 (7)
	Assistant nurse	34 (75)	53 (78)
	Care Assistant	8 (18)	10 (15)
**Years in profession**	1.5–18	26 (58)	39 (57)
	19–47	19 (42)	29 (43)
**Workplace**	Home care	20 (44)	27 (40)
	Nursing home	25 (56)	41 (60)
**Work time**	Day	30 (67)	45 (66)
	Night	6 (13)	8 (12)
	Both Day and Night	9 (20)	15 (22)
**Previous skills development in oral health**	Yes	10 (22)	13 (19)
	No	35 (78)	55 (81)
**Oral health care is included in my work duties**	Yes	41 (91)	64 (94)
	No	4 (9)	4 (6)
**Experience how the workplace manages oral healthcare**	Bad Good or very good	11 (24) 34 (76)	16 (24) 52 (76)

^a)^ AKO = Attitudes to and Knowledge of Oral health questionnaire

### Attitudes to and knowledge of oral health

No statistical significance was shown for the total score in the AKO item group Attitudes to oral hygiene (AT), except among those with work experience of under 19 years (*p* = 0.043) who had a lower mean score after (Mean = 7.11, SD = 0.389) completing the training module compared to before (Mean = 7.69, SD = 0.366), i.e., they reported a more positive attitude to oral hygiene. Specific questions were analysed (data not shown), where differences could be seen for one question related to attitudes to oral hygiene. Participants experienced that performing oral care was less practically difficult after (Mean = 2.29, SD = 0.944) completing the digital training module compared to before (Mean = 2.80, SD = 0.894, *p* = 0.002). This was shown in all sub-groups, except among persons with work experience of over 19 years.

Implementation possibilities (IP) related to oral health were ranked high on the scale as being important, both before (Mean = 15.40, SD 3.557) and after (Mean = 15.11, SD = 3.297) completing the digital training module. Still, no statistically significant differences were shown before and after the digital training module, except among participants with work experience of more than 19 years (*p* = 0.054); a lower mean score was shown after the digital training module (Mean = 14.10, SD = 0.674) compared to before (Mean = 14.73, SD = 0.790), indicating that this group experienced more difficulties with the implementation possibilities after the digital training module.

Knowledge of importance (KI) related to oral health was ranked high on the scale, i.e., as important, both before (Mean = 27.51, SD 3.441) and after (Mean = 27.22, SD = 3.376) completing the digital training module. Still, no statistically significant differences within the sub-groups before and after the digital training model were found, as shown in Table 2.

**Table 2:** Attitudes to and Knowledge of Oral health before and after the digital training module

Group of participants		Item- group	Before Mean (SD)	After Mean (SD)	*P*
**Total group**	*n* = 45				
		AT	7.82 (1.669)	7.48 (1.841)	0.090
		IP	15.40 (3.557)	15.11 (3.297)	0.133
		KI	27.51 (3.441)	27.22 (3.376)	0.361
**Workplace**					
	Home care	AT	7.75 (1.681)	7.25 (1.681)	0.322
	*n* = 20	IP	14.90 (3.740)	14.80 (3.318)	0.120
		KI	28.05 (3.119)	27.20 (3.254)	0.964
	Nursing home	AT	7.88 (1.691)	7.68 (1.973)	0.109
	*n* = 25	IP	15.80 (3.427)	15.36 (3.327)	0.667
		KI	27.08 (3.684)	27.24 (3.538)	0.245
**Age**					
	20–44 years	AT	7.70 (1.805)	7.45 (1.933)	0.390
	*n* = 24	IP	15.29 (3.688)	15.20 (3.451)	0.594
		KI	27.25 (3.790)	26.95 (3.532)	0.575
	45–67 years	AT	7.95 (0.334)	7.52 (0.388)	0.104
	*n* = 21	IP	15.52 (0.761)	15.00 (0.696)	0.074
		KI	27.80 (0.667)	27.52 (0.709)	0.510
**Year in profession**					
	1.5–18 years	AT	7.69 (0.366)	7.11 (0.389)	**0.043**
	*n* = 26	IP	15.88 (0.711)	15.84 (0.667)	0.683
		KI	27.42 (0.748)	27.03 (0.680)	0.482
	19–47 years	AT	8.00 (0.315)	8.00 (0.350)	0.951
	*n* = 19	IP	14.73 (0.790)	14.10 (0.674)	0.054
		KI	27.63 (0.676)	27.47 (0.763)	0.591

AT = Attitudes to oral hygiene (Items 1–3).

IP = Implementation Possibilities (Items 4–7).

KI = Knowledge of Importance (Items 8–13).

*P* = Exact one-tailed 𝑃 value of Wilcoxon test between baseline and after the digital training module.

***p*** **<** 0.05

No statistical significance was shown in the differences between each sub-group before and after the digital module (data not shown). Sub-analyses related to participants’ workplace, age and years in the profession showed similar results (data not shown).

### Experiences of completing the digital training module in oral health

#### Quantitative results

The overall experience of completing the digital training module in oral health (*n* = 68) was graded as ‘high’ to ‘a very high extent’ among the majority of the participants 90% (*n = 61)* as shown in Table 3. Navigation and the login process were perceived as being easy, and the time to perform the training was experienced as being well suited by the majority. The training was seen to adapt to the healthcare professionals’ work duties to a very high extent of and to provide new knowledge about oral health (96%, *n* = 65 and 99%, *n* = 67 respectively). Digital training modules were preferred rather than traditional lecture training by 47% (*n* = 32). Sub-analyses related to participants’ workplace, age and year in profession showed similar results (data not shown).

**Table 3:** Experiences of completing the digital training module in oral health

Question Score 18–72	Total *n* = 68 Mean (SD)	A very low or Low extent’ n (%)	High extent’ n (%)	To a very high extent’ n (%)
1. The instructions for the training module are easy to understand.	3.41 (0.579)	3 (4)	34 (50)	31 (46)
2. It is easy to log in to the training module.	3.21 (0.783)	11 (15)	30 (44)	27 (41)
3. The training modules interface is user-friendly.	3.22 (0.730)	8 (12)	35 (51)	25 (37)
4. Pictures and graphics are suitable for the training module.	3.54 (0.584)	3 (4)	25 (36)	40 (60)
5. It is easy to navigate the training module.	3.28 (0.688)	5 (7)	37 (54)	26 (39)
6. The training module is well structured.	3.34 (0.660)	7 (10)	31 (46)	30 (44)
7. The training module provides the opportunity to make mistakes/wrong choices and later correct them.	2.93 (0.852)	17 (25)	34 (50)	17 (25)
8. It is clear what knowledge the training module provides.	3.54 (0.584)	3 (4)	25 (37)	40 (59)
9. The training module provides knowledge about oral health.	3.75 (0.469)	1 (1)	15 (22)	52 (77)
10. The training module is experienced as instructive.	3.60 (0.550)	2 (3)	23 (34)	43 (63)
11. The training module provides useful knowledge if you work in healthcare.	3.60 (0.577)	3 (4)	21 (31)	44 (65)
12. The training module is rewarding.	3.47 (0.657)	6 (9)	24 (35)	38 (56)
13. The terminology of the training module is correct.	3.47 (0.559)	2 (3)	32 (47)	34 (50)
14. The terminology of the training module is consistent.	3.37 (0.621)	3 (4)	36 (53)	29 (43)
15. The time to carry out the training module was well suited.	3.19 (0.629)	8 (12)	39 (57)	21 (31)
16. I have sufficient prior knowledge to be able to assimilate the content of the training module.	3.47 (0.585)	3 (4)	30 (44)	35 (52)
17. Traditional training, such as lectures, is a better form of skills development than digital training modules.	2.56 (0.887)	36 (53)	20 (29)	12 (18)
18. The general assessment of the digital training module is good.	3.46 (0.584)	3 (4)	31(46)	34 (50)
Total	60.09 (7.391)	124 (10)	522 (43)	578 (47)

### Qualitative results for experiences of completing the digital training module in oral health

The conventional qualitative content analysis influenced by H-F Hsieh and SE Shannon [30] of the seven open-ended questions with free-text answers resulted in two main categories: knowledge and learning; and traditional versus digital training.

### Knowledge and learning

The participants’ free-text answers expressed that the training shed light on crucial knowledge that is easily de-prioritised in health care. One participant described it as follows:

*An important thing that highlights the importance of taking care of our older adults’ oral health and health. Unfortunately, this is often neglected.*

Previously completed training in oral health care was supplemented, and knowledge was renewed during the training. The training was instructive; nevertheless, some moments were perceived to be lengthy and were suggested to be shortened and reduced in number. Some parts were missing, such as information/knowledge about aids when brushing teeth and practical tips for performing oral health care. Not being able to ask questions was expressed as a significant shortcoming in their interaction with the module and their learning. Taking one’s own responsibility for learning was necessary, and self-reflection improved the learning when it was possible to rehearse and complete the training at their own pace and when it was most convenient. The opportunities to listen and read at the same time also improved learning. Concentration and learning increased if the training could take place in private, because the digital format requires privacy, which could be challenging to achieve in the workplace.

### Traditional vs Digital training

One aspect of the training module was comparing traditional and digital training. The participants described deficiencies in their digital competence, which made the functionality of the digital training module challenging to understand. These were reflected in comments that there were several steps to complete and about frustration with the login procedure and how these aspects were more time-consuming than the training itself. The digital form also had limitations related to the possibility of social interaction in asking questions and receiving feedback. One participant described it like this:

*Digital training: listen-see, traditional training: touch-feel-see.*

Digital training can quickly become a part of work carried out independently and not in the company of colleagues. Sharing the experience with colleagues in a traditional training with lecturers allows shared experiences in a way that the digital form did not offer. Traditional training with lecturers leads to a break from work and sometimes practical exercises that can stimulate learning, and this can be perceived as being easier to focus on than the digital form. On the other hand, the digital form was described as being calmer than traditional training with lectures, and the possibility of spreading knowledge to more colleagues was acknowledged. Opportunities were also seen to combine the digital format with more traditional training with practical exercises and the option of asking questions, or showing films and illustrations with valuable practical information. The digital form was considered to be eco-friendly and time-saving; however, more traditional training was considered easier to plan. The easy accessibility and dissemination possibilities gave the digital form opportunities for adaptations in when and how the training took place and were seen as being adaptable to healthcare settings.

## Discussion

The findings of this relatively small study indicate that healthcare professionals had similar perceptions of their attitudes to and knowledge of oral health both before and after completing the digital training module in oral health. The findings show that healthcare professionals value implementation possibilities and knowledge related to oral health. The findings also indicate that healthcare professionals with less than 19 years in the profession experience a more positive attitude to oral hygiene after completing the digital training module. However, healthcare professionals with more than 19 years in the profession experience more difficulties with implementation concerning oral health after the digital training module. Previous research describes how good functionality is a crucial factor in ensuring that technology does not hinder digital competence development [22], which can be an obstacle for healthcare professionals with more than 19 years in the profession. Therefore, healthcare professionals with less than 19 years in the profession may more easily adopt digital forms of training. Previous research also describes that healthcare professionals who receive oral health skills development have better prerequisites to perform good oral health care [19], and a lack of competence can lead to improperly performed oral health care [31, 32]. Patient safety also increases if healthcare professionals continuously engage in skills development [14], as the FoC framework also highlights. The qualitative findings indicate that healthcare professionals experienced that it is easier to perform practical oral health care after completing the digital training. Previous research has also described that interventions such as providing oral health care training can significantly improve oral health among older adults, including a lack of visible plaque and no detectable denture stomatitis [24], and one way is by offering a digital training module in oral health to healthcare professionals. Healthcare professionals in this relatively small study show that the experiences of using the digital module in oral health were overall positive, and there were few variations across the different groups. Repetition was described by the participants as being associated with increased learning, as a previously performed systematic qualitative review [22] also stated. Digital training must be incorporated systematically, and learners and educators may need additional support to fully comprehend device or app functions [22]. The strategic support necessary for digital learning will likely require procedural guidance and device training tailored for individual practice settings [22], an element that could be improved in this training module, as described by the participants in this study. As previous research [19] concludes, the participants in our study agree that healthcare professionals need continuing training to provide good oral health care for older adults. The results describe how the digital training module shows high feasibility and can be explained as a complementary pathway for training, as a previous study also shows [23]. The overall experience of completing the digital training module was positive. Still, a combination of the digital format, alongside more traditional training, with practical exercises and opportunities for asking questions or watching videos and view illustrations of valuable information were considered essential for future iterations of the module. A previous systematic review and meta-analysis also shows that interactivity, practice exercises, repetition, and feedback seem to be associated with improved learning [33]. Providing continuing training for healthcare professionals maintains a high quality of care, stimulates and motivates healthcare professionals, and provides additional knowledge for promoting good teamwork [34]. It is also crucial for the development of good FoC related to oral health care [13, 15, 16].

Based on the results, some considerations must be made. The generalizability of our results could be limited by the short study period, the relatively small group size and the specific context. Moreover the questionnaire gathering the participants’ experiences of completing the digital training module in oral health was explicitly developed for this study. No validation was performed on this questionnaire and could be seen as a weakness. However, the combination of both quanitative and qualitative information was seen of value. The data were drawn from one municipality offering health care in Sweden and therefore comprise a limited number of participants. The data also only include permanently employed healthcare professionals, which may be one of the reasons for the small group size. If temporary staff were also included, the group size would have been larger. The small context and subgroups are, consequently, one limitation of the result. Responding to the questionnaires was not a mandatory element of completing the training module and, as such, this may have contributed to the study’s low response rate. Another reason could be that answering requires additional elements in the training module and was therefore related to the structure of the training module. Another limitation is that a non-response analysis has not been performed, which may have had an influence on the interpretation of the results through, for example, selection bias. However, despite the small groups, the dataset is normally distributed in relation to its context. The classification of professional experience and age was based on the group size and may present a potential limitation, as there is a difference of between 1.5 years and 18 years of experience. Thus, the interpretation can be seen as indicative rather than interpretive in the result. Despite the relatively small number of participants in the study, they do reflect the distribution of healthcare professionals in the Swedish municipality healthcare setting, overall [35]. Statistically significant changes before and after completing the training module showed minor differences in mean values, and thus practical implications based on the results must be considered carefully. Other possible effects that may have influenced the results could perhaps be the Hawthorne effect (the assumption that people behave differently when they know that they are being observed), and learning effects, rather than just being based on the participants’ experiences of the digital training. Regarding the long-term change in attitudes to and knowledge of oral health, another post-survey should be completed by participants several months after the training has been completed to improve the results in the long term and to increase their generalizability. However, future studies need to be performed to evaluate the impact of the digital modules on *Previous research describes* attitudes to and knowledge of oral health in bigger groups of healthcare professionals working within older adult care, and also across different cultures and in different languages. It is also necessary to follow the development of the module over time, for example with focus groups/interviews. The results of this study, however, do provide knowledge to inform further investigations of the use of the digital training module over a longer period of time, which will help to increase the generalizability of the results.

## Conclusions

This study contributes to knowledge that a digital training module can be used, among Swedish healthcare professionals working within one municipality in Sweden. However, within the relatively short study time, the results revealed that the digital training module was not observed to have a significant effect on the attitudes to and knowledge of oral health in this population. The study indicates that it is easier for healthcare professionals to perform oral health care after completing a digital training module in oral health. The findings also have implications for informing the planning and development of continuous professional development in that healthcare professionals want to use digital training, but to do so in combination with practical exercises in the future to improve their attitudes to and knowledge of oral health in the best possible way.

### Electronic supplementary material

Below is the link to the electronic supplementary material.

Supplementary Material 1

## Data Availability

The data supporting this study’s findings are available by application to the corresponding author. However, restrictions apply to the availability of these data, which were used under license for the current study and are not publicly available. Datasets could be made available from the corresponding author on reasonable request.
