# Supplementary material for: Impacts on oral health attitude and knowledge after completing a digital training module among Swedish healthcare professionals working with older adults
Source: BMC Health Serv Res. 2024 Feb 7;24:174. doi: 10.1186/s12913-024-10639-3 (PMC10851574; doi:10.1186/s12913-024-10639-3)
Supplement: Supplementary file 1 — Supplementary Material 1 [file 12913_2024_10639_MOESM1_ESM.docx]

Appendix 1: Attitudes to and Knowledge of Oral health questionnaire (AKO)

The questionnaire aims to assess healthcare professionals’ attitudes to and knowledge of oral health. AKO contains thirteen statements about attitudes to and knowledge of oral health within three subject groups. Please mark statements 1–5 that fit best. It is important that you answer all thirteen statements in the form.

| **Attitudes to oral hygiene** | | | | |
| --- | --- | --- | --- | --- |
| **1.** I think it feels nasty to take care of other people’s mouths | | | | |
| Never |  |  |  | Always |
| 1 | 2 | 3 | 4 | 5 |
| **2.** I think it is practically difficult to perform oral care | | | | |
| Never |  |  |  | Always |
| 1 | 2 | 3 | 4 | 5 |
| **3.** The caregiver refuses to receive help with oral care | | | | |
| Never |  |  |  | Always |
| 1 | 2 | 3 | 4 | 5 |
| **Implementation possibilities**  What opportunities do you think you have when it comes to offering oral care to the healthcare provider you are responsible for? | | | | |
| **4**. I can take the time needed to provide oral care | | | | |
| Never |  |  |  | Always |
| 1 | 2 | 3 | 4 | 5 |
| **5**. I have enough knowledge to perform proper oral care | | | | |
| Never |  |  |  | Always |
| 1 | 2 | 3 | 4 | 5 |
| **6**. I have appropriate aids for the implementation of proper oral care | | | | |
| Never |  |  |  | Always |
| 1 | 2 | 3 | 4 | 5 |
| **7**. To caregivers who want to take care of their oral care themselves. I can give appropriate oral care advice | | | | |
| Never |  |  |  | Always |
| 1 | 2 | 3 | 4 | 5 |
| **Knowledge of importance**  What skills do you think are important for being able to perform good oral care? | | | | |
| **8**. Assistive products and oral care | | | | |
| Unimportant |  |  |  | Important |
| 1 | 2 | 3 | 4 | 5 |
| **9.** Diseases affecting the oral cavity | | | | |
| Unimportant |  |  |  | Important |
| 1 | 2 | 3 | 4 | 5 |
| **10**. Various artificial (prosthetic) dental substitutes | | | | |
| Unimportant |  |  |  | Important |
| 1 | 2 | 3 | 4 | 5 |
| **11**. What the healthy oral cavity looks like | | | | |
| Unimportant |  |  |  | Important |
| 1 | 2 | 3 | 4 | 5 |
| **12**. Oral physiological function (e.g. chewing, swallowing and speech) | | | | |
| Unimportant |  |  |  | Important |
| 1 | 2 | 3 | 4 | 5 |
| **13.** The psychosocial function of the oral cavity (e.g. appearance. well-being) | | | | |
| Unimportant |  |  |  | Important |
| 1 | 2 | 3 | 4 | 5 |
